# Retraction: High Glucose Induced Oxidative Stress and Apoptosis in Cardiac Microvascular Endothelial Cells Are Regulated by FoxO3a

**DOI:** 10.1371/journal.pone.0344069

**Published:** 2026-03-03

**Authors:** The PLOS One Editors

After this article [1] was published, concerns were raised regarding results presented in Figs 2, 6 and 7. Specifically,

- The following panels appear similar:
  - Fig 6A DAPI Control Crt siRNA, Fig 2A DAPI 6h in [2,3], Fig 2A DAPI 12h in [2,3], Fig 2A DAPI 30 mg/kg in [4], retracted in [5], when flipped horizontally, and Fig 2A DAPI 10 mg/kg in [4], retracted in [5], when flipped horizontally.
  - Fig 2A DAPI 24h, Fig 2A Merge 24h, Fig 2A ROS 24h in [2,3], Fig 2A DAPI 24h in [2–3] and Fig 2A DAPI Vehicle in [4], retracted in [5], when flipped horizontally.
  - The following panels appear to partially overlap:
  - Fig 7A Akt and Fig 2C total Akt in [6].
  - Fig 7A pFoxOa and Fig 7A BimEL in [2,3].
- There appears to be a vertical discontinuity between lanes 3 and 4 in Fig 7A Bim.

The authors did not respond to editorial request for comment on these concerns.

In light of the above concerns which call into question the integrity and reliability of [1], the *PLOS One* Editors retract this article.

All authors either did not respond directly or could not be reached.
